# The bayesian approach of factors associated with preterm birth among mothers delivered at public hospitals in Southeast Ethiopia

**DOI:** 10.3389/fpubh.2022.881963

**Published:** 2023-01-09

**Authors:** Yilkal Negesse, Gossa Fetene Abebe

**Affiliations:** ^1^College of Health Sciences, Debre Markos University, Debre Markos, Ethiopia; ^2^College of Medicine and Health Science, Mizan-Tepi University, Mizan Teferi, Ethiopia

**Keywords:** preterm birth, public hospitals, Sidama region, Ethiopia, women

## Abstract

**Background:**

Preterm birth remains the commonest cause of neonatal mortality, and morbidity represents one of the principal targets of neonatal healthcare. Ethiopia is one of the countries which accounts for the highest burden of preterm birth. Therefore, this study aimed to assess factors associated with preterm birth at public hospitals in Sidama regional state.

**Methods:**

A facility-based cross-sectional study was conducted at public hospitals in Southeast Ethiopia from 1 June to 1 September 2020. To recruit the study participants, systematic random sampling techniques were used. Data were collected using pretested structured interviewer-administered questionnaire and a checklist *via* chart review. Data were entered using EpiData version 3.1 and exported to R software version 4.0 for analysis. Then, factors associated with preterm birth among mothers were assessed based on the Bayesian statistical approach.

**Results:**

The study showed that the prevalence of preterm birth was 20.6%. Being a rural resident (AOR = 2; 95% CrI: 1.2–3.5), having no antenatal care service utilization (AOR = 2.3; 95% CrI: 1.1–4.8), hypertensive disorder of pregnancy (AOR = 3.5; 95% CrI: 1.8–6.9), birth space less than 2 years (AOR = 3.4; 95% CrI: 1.5–7.9), having premature rupture of membrane (AOR = 2.4; 95% CrI: 1.3–5.4), and physical intimate violence (AOR = 2.876; 95%CI: 1.534, 5.393) were risk factors of preterm birth. Whereas, women who had primary, secondary, and higher education levels (AOR = 0.2; 95% CrI: 0.1–0.4, AOR = 0.1; 95% CrI: 0.06–0.3, and AOR = 0.2; 95% CrI: 0.1–0.4), respectively, were preventive factors.

**Conclusion:**

Most of the risk factors of preterm birth were found to be modifiable. Community mobilization on physical violence during pregnancy and antenatal care follow-up are the ground for the prevention of preterm birth because attentive and critical antenatal care screening practices could early identify risk factors. In addition, information communication education about preterm birth prevention was recommended.

## Introduction

According to the World Health Organization, preterm birth is defined as a delivery that occurs before 37 completed weeks of gestation (1, 2). Out of the 130 million babies born each year globally, approximately 15 million are born prematurely. Of these, 60–85% are found concentrated in Africa and South Asia where health systems are difficult to access and minimum utilization of health services (3). In Ethiopia, the prevalence of preterm birth ranges from 4.4 to 31.1% (4, 5).

Preterm birth is a major healthcare problem causing over 1 million deaths of neonates annually, and high rates of morbidity and disability among survivors (6). Preterm babies predominantly suffer not only from the immediate complication of prematurity but also long-term complications, such as cerebral palsy, intellectual impairment, chronic lung disease, and vision and hearing loss (6, 7). Family, society, and the country also suffer from the economic burden of preterm birth due to longer hospital stay, neonatal intensive care, and ongoing long-term complex health needs occasioned by the resultant disabilities (7).

Ethiopia is one of the top eight countries that account for the high prevalence of preterm birth in 2014 and the top six countries that contribute nearly two-thirds of all deaths from preterm birth complications worldwide in 2016 (8). In Ethiopia, 377,000 babies are born too prematurely each year and 23,100 children under 5 years died due to direct preterm complications (9). Even if Ethiopia had achieved the Millennium Development Goal-4 (MDG-4), 2 years before the targeted year, evidence showed that the pace cannot continue as per the plan, and then neonatal mortality increased (10).

Target 3.2 of Sustainable Development Goal (SDG) 3 is used to reduce global neonatal mortality to at least as low as 12 per 1,000 live births by 2030 (11). Also, the Ethiopian government aspires to decrease neonatal mortality to 10 per 1,000 live births by 2035 (12). However, in Ethiopia, the current neonatal mortality is 30, which is too far from the target set by SDG and FMoH (13). To achieve SDG and FMoH targets, better prevention and management of preterm birth and its complications are key strategies (3). Identification of risk factors is crucial to prevent and maintain the management of preterm birth. So, to prevent and control preterm birth, it is necessary to know the determinants of preterm birth using an appropriate statistical method of analysis. The Bayesian analysis approach is one of the data analysis approach independent of the classical analysis approach and the parameters are estimated from the posterior distribution, which is the combination of the prior information and the likelihood of the data (14). A prior distribution of a parameter is the probability distribution that represents our uncertainty about the parameter before the current data are examined and the likelihood function (often simply called the likelihood) expresses how probable a given set of observations is for different values of the statistical parameters (14). However, there is no evidence that shows studies conducted at the regional level to identify determinants of preterm birth by using the Bayesian statistical approach. Nevertheless, still there is a paucity of evidence regarding factors associated with preterm birth at the country level in general and in the study area, in particular, using an advanced statistical approach. Therefore, this study aims to determine the prevalence of preterm birth and its associated factors among mothers delivered at public hospitals in Sidama regional state using the Bayesian approach.

## Materials and methods

### Study design, setting, period, and population

An institution-based cross-sectional study was conducted at public hospitals in the southeastern part of Ethiopia (Sidama regional state) from 1 June to 1 September 2020. Mothers who gave birth at public hospitals in Southeast Ethiopia (Sidama regional state) were the source population, and mothers who gave birth in the selected public hospitals during the study period were the study population. Mothers who gave live birth to singleton preterm neonate and their index neonates were included. But, mothers without a confirmed diagnosis of preterm birth due to did not remember their LNMP or have not had an early ultrasound record were excluded from the study. Similarly, medically induced preterm births are also excluded from this study.

### Sample size determination and sampling technique

The sample size was determined using the single population proportion formula $n\geq {\left[\frac{Z_{\frac{\alpha }{2}}^{2}}{\varepsilon^{2}}\right]}^{*}P\left(1-p\right)$ (15) by considering the proportion of preterm birth (*P* = 29.6%) (16), 95% confidence level (Z = 1.96), 5% of marginal error (W = 0.05), 10% non-response rate, and design effect of 2. After adding a 10% non-response rate, the required final sample was 704.

A multistage sampling technique was employed. Out of three general and 14 primary hospitals, one general and three primary hospitals, respectively, were randomly selected. In addition, there was only one comprehensive hospital located in the region and it was included in the study. The total sample size was proportionally distributed to each hospital based on an average number of deliveries recorded in the most recent quarterly report of each health facility before the study period. Finally, the study participants were recruited using a systematic random sampling technique.

### Operational definitions

**Early ultrasound record:** Ultrasound result is taken until 22 weeks of gestational age (17, 18).

**Physical intimate violence**: It is defined as any act of harm to women physically by the current or former intimate partner or husband (19).

**Hypertensive disorders of pregnancy**: It is defined as systolic blood pressure (SBP) of 140 mmHg or more or diastolic blood pressure (DBP) of 90 mmHg or more on 2 or more consecutive occasions during pregnancy (20).

### Data collection tools, procedures, and techniques

Data were collected through face-to-face interviews using a standardized, structured, and pretested questionnaire and a chart review checklist. The questionnaire and checklist were adapted from other similar studies with some contextual modifications (19, 21). It contains sociodemographic characteristics, obstetric factors, preexisting medical factors, fetal factors, and physical intimate partner violence. A checklist was used to collect data from the medical record and actual measurements.

The gestational age was established based on a certain last menstrual period (LMP) date and/or early pregnancy ultrasound determined date (up to and including 22 completed weeks of gestation). When the LMP and U/S dates had not been correlated, U/S for gestational age assessment was taken in accordance with the recommendation of the American College of Obstetricians and Gynecologists (ACOG) (17, 18, 22). Those mothers with neither reliable LMP nor early pregnancy U/S date for GA estimation had been excluded. Data were collected by 10 trained BSc (Bachelor of Science) midwives who had been working in the delivery ward. Five BSc midwives were trained and supervised the data collection.

### Data quality assurance

The questionnaire was prepared first in English and translated into the local language Amharic and Sidaamu Afoo, and finally, retranslated back to English by a language expert to increase accuracy. Before conducting the study, the questionnaire was pretested on 10% of the sample. Based on the pretest, an appropriate modification was made. The 1-day training was provided for data collectors and supervisors.

### Data processing and analysis

The collected data were checked for completeness, entered into EpiData version 3.1, coded, cleaned, and exported to R software version 4.0 for further analysis. After the data were cleaned and coded, descriptive measures, such as frequency, percentage, graphs, and tables, were used to characterize the study population. The relationship between the dependent and independent variables was assessed using the binary logistic regression model based on the Bayesian statistical analysis approach. The dependent variable (preterm birth) is represented by *Y*_*i*_ = $\{\begin{array}{c} expriancing\;pre\;term\;birth\\ not\;expriancing\;pre\;term\;birth\; \end{array}$, which is a categorical type of data. Thus, to estimate the parameters of the variable, binary logistic regression analysis based on the Bayesian approach was done using Brms R-package. Since we do not have prior information, we used vague prior distribution to determine the posterior distribution. To estimate regression coefficients, we used beta distribution (1, 1), and to estimate the variance, we used gamma distribution (0.001, 0.001). We also used chains = 2, initials (the starting values of the iterations) = 0, iteration = 10,000, cores (specifies the number of cores used for the algorithm) = 2, warmup (number of iterations that was discarded) = 1,000, and adapt delta (controls divergent transition) = 0.95 to estimate the estimates of the parameters from the posterior distribution.

Then, Hamiltonians Monte Carlo (HMC) method was performed to simulate direct draws from the complex posterior distribution. Therefore, since the iteration convergence is fast, we used No-U-Turn Sampler (NUTS). Finally, summary statistics were carried out from the posterior distribution after the model was converged, and the 95% credible interval was used for the test of significance. The empirical results obtained from a given HMC analysis are not reliable until the chain has reached its stationary distribution. Therefore, to monitor the convergence of the algorithm, we used the most popular assessment methods in which Rhat = 1, Bulk_ESS and Tail_ESS were greater than 1,000, density plots were smooth, and chains of the time series plots were mixed well.

## Results

### Frequency and percentage distribution of preterm birth

The prevalence of preterm birth among women delivered in the Sidama region of Southeast Ethiopia public hospitals was 20.4%.

A total of 47.6% of women who experienced hypertensive disorder during pregnancy had a preterm birth. In addition, 19.3% of women who had premature rupture of the membrane experienced preterm birth (see Table 1).

**Table 1:** Frequency and percentage distribution of preterm birth by different variables among mothers who delivered at public hospitals in Sidama regional state, Southeast Ethiopia, 2020.

**Variables**	**Categories**	**Preterm birth**			
		**No**		**Yes**	
		**Frequency**	**Percentage**	**Frequency**	**Percentage**
Age	≤ 19	79	15.1%	28	21.2%
	20–34	214	40.9%	69	52.3%
	≥35	230	43.9%	35	26.5%
Marital status	Married	470	89%	111	82.2%
	Unmarried	58	11%	24	17.8%
Residence of respondent	Urban	391	74.1%	76	56.3%
	Rural	137	25.9%	59	43.7%
Educational status of women	No formal education	135	25.6%	71	52.6%
	Primary school	164	31.1%	34	25.2%
	Secondary school	118	22.3%	19	14.1%
	Diploma and above	111	21%	11	8.1%
ANC follow up	Yes	461	87.3%	94	69.6%
	No	67	12.7%	41	30.4%
Number of family member	< 5	50	9.5%	18	13.3%
	≥5	478	90.5%	117	86.7%
Parity	< 3	8	1.5%	9	6.7%
	≥3	520	88.5%	126	93.3%
Advised about danger sign of pregnancy	Yes	500	94.7%	115	85.2%
	No	28	5.3%	20	14.8%
hypertensive disorders of pregnancy	No	484	91.7%	96	71%
	Yes	44	8.3%	39	29%
Premature rupture of membrane	No	500	94.7%	106	78.5%
	Yes	28	5.3%	29	21.5%
Urinary tract infections	No	498	94.3%	91	67.4%
	Yes	30	5.7%	44	32.6%
Hgb of mother at booking	≤ 10	59	11.2%	25	18.5%
	≥11	469	88.8%	110	81.5%
HIV status of Mother	Non-reactive	509	96.4%	121	89.6%
	Reactive	19	3.6%	14	10.4%
Intimate partner violence	No	500	94.7%	94	69.6%
	Yes	28	5.3%	41	30.4%

### Binary logistic regression based on the Bayesian approach

As shown in Table 2, this model shows that the Rhat value is one and all effective sample sizes (both Bulk_ESS and Tail_ESS) are greater than 300 times the number of chains. In addition to Rhat and effective sample size, the density plots were smooth and also chains of the trace plots were mixed well. Therefore, the model converged well. Since the model is converged, all estimates obtained from this model are reliable. Thus, all interpretations and inferences were made based on this model.

**Table 2:** Relationship analysis for preterm birth and predictors based on the Bayesian approach in Southeast Ethiopian women in 2020.

**Variables**	**Characteristics**	**Estimates**	**SE**	**AOR**	**95%CrI of AOR**		**Rhat**	**Bulk_ ESS**	**Tail_** **ESS**
					**L-CrI**	**U-CrI**			
β0 intercept		−0.86	0.66	0.42	0.11	1.56	1	8,422	6,537
Age	≤ 19	0.83	0.38	1.28	0.98	2.4	1	6,432	7,231
	20–34 (ref)						1		
	≥35	0.61	0.33	0.92	0.53	1.66	1	6,648	5,882
Marital status	Unmarried	0.11	0.38	1.11	0.53	2.3	1	8,300	6,530
	Married (ref)								
Residence	Urban (ref)								
	Rural^*^	0.69	0.29	2	1.2	3.5	1	8,180	6,129
Educational status	Not formal (ref)								
	Primary^*^	−1.6	0.34	0.2	0.1	0.4	1	6,635	6,050
	Secondary^*^	−2	0.4	0.1	0.06	0.3	1	7,209	5,949
	Higher^*^	−1.83	0.47	0.2	0.1	0.4	1	7,271	6,767
Number of family member	< 5 (ref)								
	≥5	0.06	0.49	1.1	0.4	2.8	1	7,918	6,312
Parity	≥3	−0.34	0.29	0.7	0.4	1.2	1	8,425	6,469
	< 3 (ref)								
Birth interval in year^*^	< 2 year	1.24	0.42	3.4	1.5	7.9	1	9,054	6,112
	≥2 year (ref)								
Antenatal care follow up^*^	No	0.85	0.37	2.3	1.1	4.8	1	7,368	6,258
	Yes (ref)								
Fist antenatal care started	< 16 weeks (ref)								
	≥16 weeks	0.08	0.32	1	0.6	2	1	7,733	5,995
Danger sign of pregnancy advised	No	0.57	0.42	1.8	0.8	4	1	8,239	6,152
	Yes (ref)								
hypertensive disorders of pregnancy^*^	Yes	1.24	0.36	3.5	1.8	6.9	1	8,662	6,152
	No (ref)								
Premature rupture of membrane^*^	Yes	0.88	0.42	2.4	1.3	5.4	1	8,065	6,135
	No (ref)								
Hgb of mother at booking	< 11	0.21	0.36	0.8	0.4	1.7	1	8,828	6,607
	≥11 (ref)								
Urinary tract infections	Yes	1.1	0.35	3	1.5	5.9	1	8,785	6,642
	No (ref)								
HIV status of Mother	Positive	0.41	0.5	1.5	0.5	4	1	9,477	6,421
	Negative (ref)								
Sex of neonate	Male (ref)								
	Female	0.16	0.28	1.2	0.7	2	1	9,035	6,443
Intimate partner violence^*^	Yes	1.28	0.37	3.6	2	7	1	7,775	6,345
	No (ref)								

ref, reference category;

* significant at 95% CrI; SE, Standard Error; AOR, Adjusted Odd Ratio; CrI, Credible Interval; Hgb, Hemoglobin.

## Discussion

Even though there have been advancements in perinatal medicine and feto maternal units, preterm birth remains the leading cause of neonatal mortality and morbidity, leading to the first place for neonatal intensive care unit admission and longer hospital stay (23, 24). This study aimed to assess the factors of preterm birth among mothers who gave birth at public hospitals in Southeast Ethiopia.

The magnitude of preterm birth in this study was consistent with the previous findings conducted in Bangladesh at 22.3% (25), Nigeria at 16.9% (26), Kenya at 18.3% (27), and Jimma at 25.9% (28). However, the finding of this study was higher than a study conducted in Brazil at 12.5%(29), Northwest Ethiopia at 12.8% (30), and northern Ethiopia at 13.3% (31). The discrepancy might be due to sociodemographic and economic variations of the study participants. Most of the participants in the current study were rural dwellers compared with the previous study that claimed an increased risk of preterm birth.

Of all the factors included in the model, being a rural resident, educational status of the mother, birth interval, ANC follow-up, hypertensive disorders of pregnancy, the premature rupture of membrane, and physical intimate violence were significantly associated with preterm birth in southern Ethiopia (Sidama regional state). The odds of delivering preterm babies among mothers who lived in rural areas were more likely than among urban residents. This finding is similar to the study conducted in the Amhara region (19), and Axum and Adwa town public hospitals (31). This might be explained by women who reside in rural areas are more likely to be exposed to hard physical work such as farming, which, in turn, increases the risk of preterm delivery. Also, in rural areas, there is no access to healthcare facilities, transportation, and improved water sources (32). Therefore, they travel a long distance to access them. This might lead them to experience preterm birth. The odds of having preterm birth among mothers who did not attend formal education were more likely than educated women. This result is supported by a cohort study conducted in Europe (33). The study revealed that mothers who did not utilize antenatal care during their current pregnancy were more likely to deliver preterm babies as compared to mothers who utilized ANC. This result is consistent with the study conducted in Dodola town hospitals, Southeast Ethiopia (34), Kampala, Uganda (35), and a systematic review in East Africa (36). This might be because women who had no ANC follow-up could miss information that is important to prevent, identify, refer, and treat preterm birth promptly in health facilities.

This study depicts that the odds of preterm delivery among mothers who experienced hypertensive disorders of pregnancy were more likely than mothers who have not experienced hypertensive disorders of pregnancy. This finding is in line with a study conducted in Tigray (37), Jimma (21), and Kenyatta national hospitals (38). This might be because hypertension decreases the uteroplacental blood and nutrients transfer, which leads to intrauterine growth restriction and/or early placenta dysfunction that cause preterm delivery (39).

The odds of preterm delivery among mothers who experienced birth spacing <2 years were more likely as compared to those who spaced more than 2 years. This finding is in line with a study conducted in Jimma (21), Northern Ethiopia (40), Axum and Adwa town (31), and Amhara regions (19). This might be due to mothers having short inter-pregnancy intervals cannot recover from the biological stress imposed by the preceding pregnancy resulting in the reduction of macronutrients supplementation in the maternal body, folate depletion, cervical insufficiency, vertical transmission of infections, incomplete healing of uterine scar and abnormal remodeling of endometrial blood vessels, anemia, and maximizing the risk of certain other factors achieving pregnancy outcomes (41, 42).

The odds of mothers who experienced physical intimate violence during pregnancy were more likely to deliver preterm babies than mothers who did not experience physical intimate violence. This finding is in agreement with a study conducted in Iran (43), Vietnam (44), and Tanzania (45). This might be due to physical violence during pregnancy affecting premature delivery because of physical trauma upon the abdomen, uterus, and post-trauma-induced stress, which leads to premature onset of labor related to either direct effect or due to corticotrophin-releasing hormone (CRH) (46, 47). This finding has not been supported by a study conducted in Canada (48) and the Amhara region (19). This difference could be explained by a difference in the methods used for data analysis, the discrepancy in the measurement of physical intimate violence, and the difference in the sociodemographic characteristics of respondents.

### Strengths and limitations of the study

This study identified several factors associated with preterm birth using an appropriate method of statistical analysis. But, there were some limitations to this study. The study participants might not remember (recall bias) their exact LNMP. So, to solve this problem, we used ultrasound records. The other limitation of the study was the prevalence of preterm birth in the Sidama regional state might be biased by referrals from the neighboring southwest Arusi and Oromia regional state hospitals. Thus, to alleviate this problem, we excluded women who have referral papers in their chart from the study.

## Conclusion

Preterm birth had been a major public health problem in the study area for perinatal care, and thus deserves priority attention. There were many factors interwoven to affect the occurrence of preterm birth. Preterm birth is more likely to occur in women living in rural areas, with no ANC follow-up, narrow birth spacing, and suffering from physical violence during pregnancy. Whereas, women who attend primary education and above were preventive factors. Thus, efforts should be intensified to alleviate the identified risk factors to minimize the burden of preterm birth in the study setting, Southeast Ethiopia. Community mobilization on physical violence during pregnancy and ANC follow-up are the ground for the prevention of preterm birth because attentive and critical ANC screening practices could early identify the risk factors. Further community-based longitudinal (cohort) studies might explore additional determinants of preterm birth.

## Data availability statement

The original contributions presented in the study are included in the article/supplementary material, further inquiries can be directed to the corresponding author.

## Ethics statement

Ethical clearance was obtained from the Institutional Review Board (IRB/202/12) at the College of Medicine and Health Sciences of Hawassa University. An official letter of cooperation was obtained from the Department of Midwifery to respective hospital administrators. Informed written permission was obtained from each hospital administrator. After the purpose and objective of the study have been explained, written consent was obtained from each study participant. Participants were informed that participation was on a voluntary basis and can withdraw from the study at any time if they were not comfortable with the questionnaire. In order to keep confidentiality, information was maintained throughout by excluding names or personal identifiers in the questionnaire.

## Author contributions

YN conceived the study, analyzed the data, and wrote the first draft of the manuscript. GA supervised the study, methods, and proofread the manuscript. All the authors read and approved the final version of the manuscript.
